# Identification of metabolic pathways contributing to ER^+^ breast cancer disparities using a machine-learning pipeline

**DOI:** 10.1038/s41598-023-39215-1

**Published:** 2023-07-26

**Authors:** Ashlie Santaliz-Casiano, Dhruv Mehta, Oana C. Danciu, Hariyali Patel, Landan Banks, Ayesha Zaidi, Jermya Buckley, Garth H. Rauscher, Lauren Schulte, Lauren Ro Weller, Deanna Taiym, Elona Liko-Hazizi, Natalie Pulliam, Sarah M. Friedewald, Seema Khan, J. Julie Kim, William Gradishar, Scott Hegerty, Jonna Frasor, Kent F. Hoskins, Zeynep Madak-Erdogan

**Affiliations:** 1 Division of Nutritional Sciences, University of Illinois, Urbana-Champaign, Urbana, IL USA; 2 Food Science and Human Nutrition Department, University of Illinois, Urbana-Champaign, Urbana, IL USA; 3 Division of Hematology/Oncology, University of Illinois at Chicago, Chicago, IL USA; 4 School of Public Health, University of Illinois at Chicago, Chicago, IL USA; 5 Robert H. Lurie Cancer Center of Northwestern University, Chicago, IL USA; 6 Northwestern Memorial Hospital, Chicago, IL USA; 7 Northwestern University Feinberg School of Medicine, Chicago, IL USA; 8 Northeastern Illinois University, Chicago, IL USA; 9 Department Physiology and Biophysics, University of Illinois at Chicago, Chicago, IL USA; 10 Department of Biomedical and Translational Sciences, Carle Illinois College of Medicine, Urbana, IL USA; 11 Carl R. Woese Institute for Genomic Biology, University of Illinois, Urbana-Champaign, Urbana, IL USA; 12 Cancer Center at Illinois, 1201 W Gregory Dr, Urbana, IL 61801 USA

**Keywords:** Cancer metabolism, Endocrine cancer, Tumour biomarkers

## Abstract

African American (AA) women in the United States have a 40% higher breast cancer mortality rate than Non-Hispanic White (NHW) women. The survival disparity is particularly striking among (estrogen receptor positive) ER^+^ breast cancer cases. The purpose of this study is to examine whether there are racial differences in metabolic pathways typically activated in patients with ER^+^ breast cancer. We collected pretreatment plasma from AA and NHW ER+ breast cancer cases (AA n = 48, NHW n = 54) and cancer-free controls (AA n = 100, NHW n = 48) to conduct an untargeted metabolomics analysis using gas chromatography mass spectrometry (GC–MS) to identify metabolites that may be altered in the different racial groups. Unpaired t-test combined with multiple feature selection and prediction models were employed to identify race-specific altered metabolic signatures. This was followed by the identification of altered metabolic pathways with a focus in AA patients with breast cancer. The clinical relevance of the identified pathways was further examined in PanCancer Atlas breast cancer data set from The Cancer Genome Atlas Program (TCGA). We identified differential metabolic signatures between NHW and AA patients. In AA patients, we observed decreased circulating levels of amino acids compared to healthy controls, while fatty acids were significantly higher in NHW patients. By mapping these metabolites to potential epigenetic regulatory mechanisms, this study identified significant associations with regulators of metabolism such as methionine adenosyltransferase 1A (MAT1A), DNA Methyltransferases and Histone methyltransferases for AA individuals, and Fatty acid Synthase (FASN) and Monoacylglycerol lipase (MGL) for NHW individuals. Specific gene Negative Elongation Factor Complex E (NELFE) with histone methyltransferase activity, was associated with poor survival exclusively for AA individuals. We employed a comprehensive and novel approach that integrates multiple machine learning and statistical methods, coupled with human functional pathway analyses. The metabolic profile of plasma samples identified may help elucidate underlying molecular drivers of disproportionately aggressive ER+ tumor biology in AA women. It may ultimately lead to the identification of novel therapeutic targets. To our knowledge, this is a novel finding that describes a link between metabolic alterations and epigenetic regulation in AA breast cancer and underscores the need for detailed investigations into the biological underpinnings of breast cancer health disparities.

## Introduction

Breast cancer mortality rates in African American (AA) women are 40% higher than in Non-Hispanic White (NHW) women^1^, stemming from complex and multifaceted factors^2^. AA women experience earlier disease onset, exhibit biologically aggressive tumor phenotypes^1–3^, present with higher stages at diagnosis (4), experience increased likelihood of distant metastases^4^*,* and have lower survival rates post-diagnosis^4–6^. These disparities are particularly pronounced in individuals with estrogen receptor-positive (ER^+^) breast cancer^1^. While ER^+^ breast tumors typically respond favorably to endocrine therapy, leading to better outcomes, this improvement is not equally reflected in the overall survival rates of AA individuals. Thus, advancements in treatment that have significantly reduced breast cancer mortality overall, have not yielded equitable survival equally for all racial groups. Early studies suggested that the unfavorable outcomes among AA women was attributed to disparities in socioeconomic status, education and healthcare access^7–11^. Comprehensive meta-analysis at the population level confirmed that AA women continue to experience significantly higher breast cancer mortality rates compared with NHW women even after considering socioeconomic status^12,13^. Department of Defense studies furthered revealed that despite equal healthcare access and treatment protocols, AA women still faced poorer breast cancer outcomes than NHW women^14,15^. Additional investigations, adjusting for hormone receptor subtype, area-level factors, socioeconomic status and healthcare access consistently demonstrated significantly higher breast cancer mortality rates among AA women compared to NHW women^16^. Recent epidemiological findings from Chicago demonstrated that AA women with ER^+^ breast cancer faced a hazard of death that was 4–5 times higher than their white counterparts even after accounting for stage at diagnosis, tumor grade, and treatment^1^. Breast cancer mortality associated with ER+ cancers was higher in AA women treated in non-accredited hospitals, and from lower socioeconomic status neighborhoods^17^.

A deeper look into the biological mechanisms that affect tumor aggressiveness is crucial to understanding this disparity. The human metabolome reflects an individual metabolic state, which is influences by external factors like diet and environmental exposures. Usually, tumors exhibit increased demands for various nutrients in a frequently nutrient-poor environment to support sustained growth and proliferation. These alterations in demands and use of intracellular and extracellular metabolites are characteristic of cancer metabolic reprogramming, as it is well known that intermediate metabolites and products can act as signaling or epigenetic modulators^18–21^. Metabolomics profiling offers insights into cell function by directly influencing the phenotype of cancer cells^22^. In the context of breast cancer, this approach has successfully utilized to identified cancer risk factors^23–25^, prognostic indicators^26–28^, and early detection biomarkers^26,29–31^. Moreover, it has revealed disease-specific metabolic patterns and their associations with clinical outcomes^32,33^. Notably, metabolomics profiling has also demonstrated variations in metabolite levels across different racial groups^34–37^. These differences persists even after accounting for baseline characteristics such as lifestyle factors, health conditions, medications, and socioeconomic status.

Unfortunately, few studies have examined the role of circulating metabolites and the associated regulatory pathways that can help explain more aggressive tumor phenotypes and worsened disease outcomes in AA women. If racial differences exist in these associations, it may enhance understanding of the main biological and socioeconomic factors that drive racial disparities in ER^+^ breast cancer. Studying metabolic alterations in AA women, can provide insights into lifestyle, barriers to health access, dietary factors, and other contributors to the disparity in mortality in this racial group, which are often challenging to measure.

We previously used metabolomics analyses coupled with machine learning analysis to identify cancer-associated biomarkers^38^. Using a combination of multiple machine learning methods, statistics, and visualization coupled with human pathway analyses, our current study aims to identify differences in metabolic signatures and associated metabolic mechanisms perturbed in AA patients with ER^+^ breast cancer to help explain why they are more likely to die from the disease than their white counterparts are.

## Methods

### Study population

African American (AA) and Non-Hispanic White women (NHW) aged 20–79 years, with a new diagnosis of (American Joint Committee of Cancer) AJCC stage I–III, ER^+^ breast cancer were recruited from 3 hospitals in Chicago, IL between 2018 and 2019. This study was approved by the University of Illinois, Chicago Institutional Review Board (IRB protocol number 2017-1029). An informed consent was obtained from all subjects. All methods were performed in accordance with the relevant guidelines and regulations. Healthy AA and NHW women without breast symptoms or a personal history of breast cancer who presented for a screening mammogram at corresponding mammography centers were recruited as control subjects. Only control subjects whose screening mammogram was negative (BI-RADS category 1-2) were included in the analytic study population. Non-fasting whole blood was collected from AA and NHW breast cancer cases prior to breast surgery or any other breast cancer treatment. Control subjects donated a blood specimen at the time of study enrollment. Demographic and tumor pathologic characteristics are described in Table 1. Whole blood collected in serum separator tubes was allowed to clot for 40 min at room temperature, and plasma was separated by centrifugation within 1 h of collection (1680*g* for 10 min at room temperature in a horizontal rotor, tabletop centrifuge). Aliquots of plasma were immediately frozen at − 20 °C, and transferred to − 80 °C within 4 weeks. All samples were analyzed after a single freeze–thaw cycle.

**Table 1:** Demographic and tumor pathologic characteristics.

	Cases			Controls		
	AA	NHW	p value	AA	NHW	p value
	(%)	(%)		(%)	(%)	
Number of participants	49	54	0.121	105	45	0.1971
Mean age, (years)	60.5	56.8		55.5	58.1	
Missing (n)	0	0		2	0	
Mean body mass index (kg)	31.6	29.4	0.10^a^	32.6	28.2	0.002^a^
Missing (n)	0	0		2	1	
Tumor grade						
Low	15(33)	13(24)	0.89^b^	–	–	
Intermediate	22(48)	31(57)				
High	9(20)	10(19)				
Missing (n)	3	0		–	–	
AJCC stage						
1	19(43)	39(75)	0.0023			
2	19(43)	12(23)				
3	6(14)	1(2)				
Missing (n)	5	2				

*AA* African American, *NHW* non-Hispanic White, *AJCC* American Joint Committee on Cancer.

^a^Student’s t-test.

^b^Chi squared test for grade 1/2 vs. grade 3.

^c^Fisher’s Exact test.

### Metabolomics analysis

#### Sample preparation

To precipitate protein, 200 µL of thawed plasma was mixed with 1 mL isopropanol, acetonitrile, H2O 3:3:2 mix. The mixture was vortexed for 10 s and kept at − 20 °C for further processing. After spinning down, 600 µL of the supernatant were transferred to a separate Eppendorf tube and submitted to UIUC metabolomics core for a GC/MS whole metabolite profiling.

### Mass spectrometry

A gas chromatography/mass spectrometry (GC/MS) based metabolic profiling was performed to detect and quantify the metabolites in plasma. Supernatants were collected by centrifugation (5 min at 15,000*g*), dried and derivatized with 50 μL of 40 mg/mL methoxyamine hydrochloride in pyridine (Sigma-Aldrich, MO, USA) for 60 min at 50 °C, then with 50 μL MSTFA + 1%TMCS (Thermo, MA, USA) at 70 °C for 120 min, and following 2-h incubation at room temperature. 50 μL of the internal standard (hentriacontanoic acid, 1 mg/mL) was added to each sample before derivatization. Metabolite profiles were acquired using a GC–MS system (Agilent Inc, CA, USA) consisting of an Agilent 7890 gas chromatograph, an Agilent 5975 MSD and an HP 7683B autosampler. Gas chromatography was performed on a ZB-5MS (60 m × 0.32 mm ID and 0.25 μm film thickness) capillary column (Phenomenex, CA, USA). The inlet and MS interface temperatures were 250 °C, and the ion source temperature was adjusted to 230 °C. An aliquot of 1 μL was injected with the split ratio of 10:1. The helium carrier gas was kept at a 2 mL/min constant flow rate. The temperature program was: 5-min isothermal heating at 70 °C, followed by an oven temperature increase of 5 °C/min to 310 °C and a final 10 min at 310 °C. The mass spectrometer was operated in positive electron impact mode (EI) at 69.9 eV ionization energy at m/z 30–800 scan range.

### Spectra and data analysis

The spectra of all chromatogram peaks were evaluated using the AMDIS 2.71 (NIST, MD, USA) software using a custom-built database with 460 unique metabolites. All known artificial peaks were identified and removed before data mining. All data were normalized to the internal standard hentriacontanoic acid, 1 mg/mL in each chromatogram, and the cell’s dry weight (DW) to compare samples. The instrument variability was within the standard acceptance limit (5%).

### Data processing

As part of data preprocessing, metabolites missing over 40% of data were removed. Remaining missing data values were completed using a trained data imputation algorithm using the available data. The imputation performance was tested one metabolite after another by removing one column and doing imputation on them. The imputation performance was evaluated for each specific metabolite considering R square metric. This process was repeated for each metabolite in the data set, removing and imputing them individually. Metabolites with R^2^ < 0.3, that indicated poor imputation performance were removed. To explore which metabolites (these are referred as features) are indicators of cancer and in each group, the data was sorted as African American (AA) data set and Non-Hispanic White (NHW) data set.

### Statistical analyses

In order to compare the metabolite levels between cases and controls in each race group, we performed unpaired t-tests between cases and controls for each metabolic feature, separated by race groups. Differential metabolites were identified by adjusting the p values for multiple testing at a False discovery Rate (FDR) threshold of 0.05. A (*) indicates p value (p < 0.05) considered statistically significant. p value is reported as two-tailed p value, and standard error is included. All p values are corrected for multiple testing using FDR original method of Benjamini and Hochberg. R.4.02, and Graphpad Prism 9.2.0 software were used for statistical analyses (t-tests) (GraphPad Software Inc., La Jolla, CA, USA, www.graphpad.com).

### Feature selection and prediction

To assess which features (metabolites) were associated with breast cancer in the cohort, we integrated a combination of machine learning feature selection and prediction algorithms. Algorithms used for feature selection included: Boruta test and Recursive Feature Elimination (RFE). Boruta algorithm identifies important features by creating shuffled shadow copies of all features and assigning threshold to each features. It selects the maximum threshold from the shadow features and applies it to the original features. Features that surpass this threshold are chosen. The algorithm iterates until all features are either confirmed or rejected as features of importance. Recursive Feature Elimination (RFE) in other hand begins with building a model on all predictors and calculating an importance score for each. The least important predictors are then removed, the model is rebuilt, and the importance scores are recalculated.

**Figure 1:**
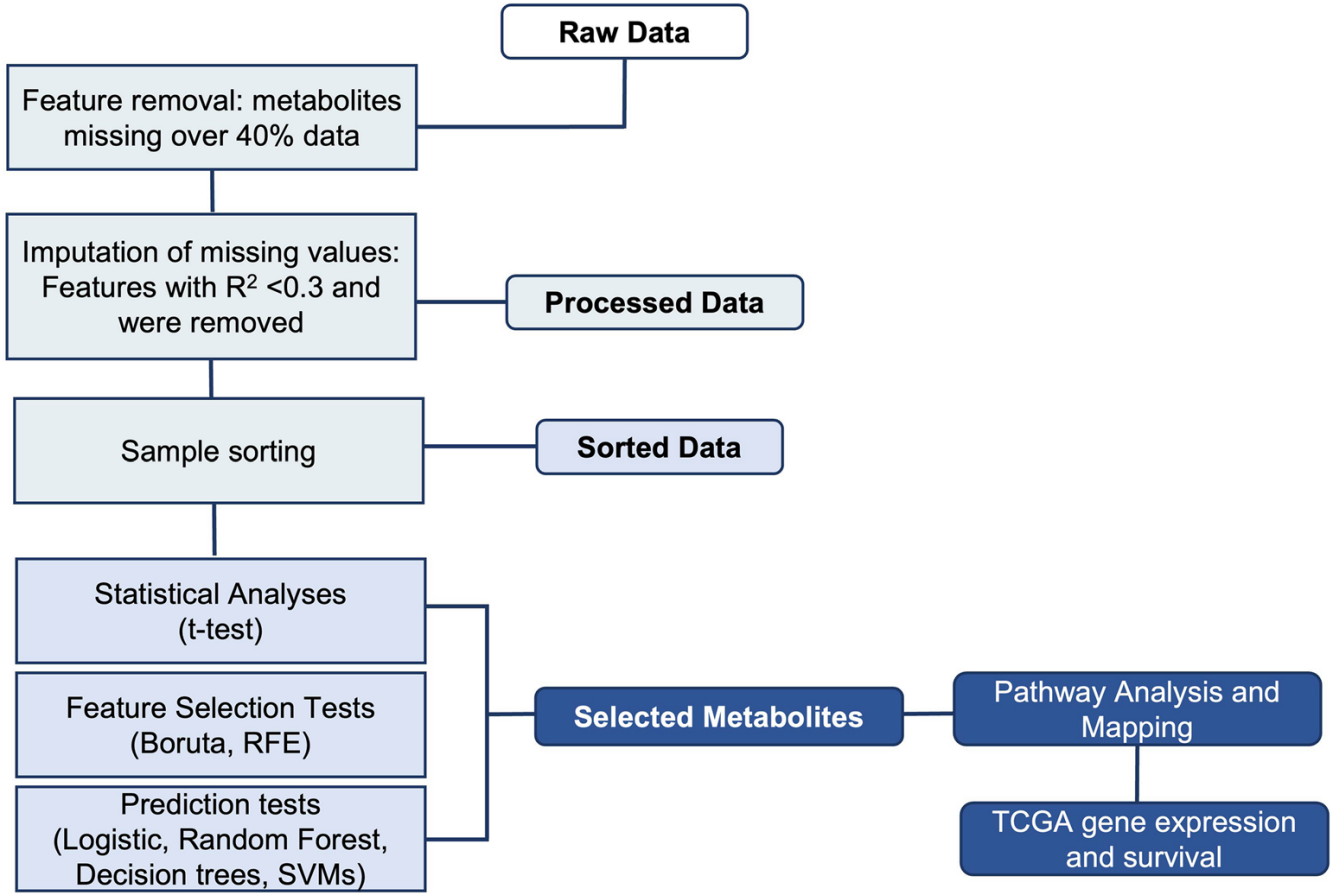
The structure of the pipeline and different steps are shown. The method consist of data quality check data sorting by race, statistical analyses, feature selection and classification tests, pathway analysis and evaluation in gene expression and survival in TCGA cohort of selected genes associated to metabolites mapped.

We explored the performance of each feature selection and classification performance method by comparing the AUC values obtained using each method combination. A complete diagram of the pipeline used is described in Fig. 1. Predictive approaches used included: Decision Tree, Random Forest, Logistic Regression, and Support Vector Machine (SVM). Boruta, caret, tidyverse, readxl, e1071, pROC, tree, randomForest R packages were used. Parameters used on each test are reported in Table 2.

**Table 2:** Parameters used in each method.

	Parameters
Feature selection algorithm	
Boruta	Default parameters
Recursive feature elimination	{functions: rfFuncs, method: repeatedcv, repeats: 5, verbose: FALSE}
Algorithm	
Random forests	{n_estimators: 100, criterion: gini, min_samples_split: 2, max_features: auto}
Decision trees	Default parameters, {criterion: gini, splitter: best, min_samples_split: 2}
Logistic regression	Default parameters, {family: binomial}
Support vector machine (SVM)	{kernel: linear, gamma: (depends on the data) (between 0.000001 and 0.1), cost: (depends on the data) (between 0.1 and 10)}

### Performance evaluation

Performance metric statistics were gathered, including Mathews Correlation Coefficient (MCC) and F1 score, as well as receiver operator characteristic and area under the curve (ROC, AUC) curves for each combination of feature selection and prediction method to visualize classification performance (true positive rate vs. false positive rate). We used the receiver operating characteristic curve (ROC) for each feature selection and prediction method as a quantitative performance metric. We further analyzed the performance of the combination methods by reporting F score statistics and MCC (Table 3). Once selected the best feature selection/prediction model combination, the top features of importance were identified. Their relative influence on each model (in %), in terms of Mean Decrease Gini were computed. These features were further explored for pathway analysis.

**Table 3:** Performance metrics.

Race	Feature selection model	Prediction method	TPR (sensitivities)	FPR	MCC	F1
AA	Boruta	Decision tree	0.7	0.166667	0.526789	0.682927
AA	Boruta	Random forest	0.909091	0.215686	0.559715	0.625
AA	Boruta	Logistic	0.733333	0.212766	0.471053	0.611111
AA	RFE	Random forest	0.833333	0.22	0.512001	0.606061
AA	RFE	SVM	0.8	0.25	0.427428	0.516129
AA	RFE	Decision tree	0.368421	0.176471	0.213946	0.4375
AA	Boruta	SVM	0.857143	0.272727	0.390796	0.428571
AA	RFE	Logistic	0.75	0.277778	0.334493	0.413793
NHW	Boruta	Random forest	0.8	0.285714	0.515515	0.761905
NHW	RFE	Logistic	0.736842	0.285714	0.450564	0.717949
NHW	Boruta	Logistic	0.647059	0.277778	0.370494	0.666667
NHW	Boruta	Decision tree	0.631579	0.4375	0.194079	0.631579
NHW	RFE	Decision tree	0.631579	0.4375	0.194079	0.631579
NHW	RFE	Random forest	0.833333	0.22	0.512001	0.606061
NHW	RFE	SVM	0.714286	0.428571	0.280976	0.606061
NHW	Boruta	SVM	0.611111	0.470588	0.140984	0.594595

Comparison of the performance of various combinations of feature selection and classification methods.

### Pathway analysis

To identify the most relevant pathways involved in each set (AA and NHW) a pathway analysis was performed using Metaboanalyst 4.0 Pathway Analysis Module. This module combines pathway enrichment analysis with pathway topology analysis. It uses high-quality KEGG metabolic pathways as the backend knowledge base and integrates many well-established (i.e., univariate analysis, over-representation analysis) methods, as well as novel algorithms and concepts (i.e., Global Test, GlobalAncova, network topology analysis) into pathway analysis. This analysis was limited to all metabolic features selected with the combination of Boruta test feature selection and Random Forest classification and that were consistent in the rest of the feature selection methods (Table 4A,B).

**Table 4:** (A) Feature importance ranking with mean decrease Gini index calculated by the random forest (RF) algorithm for AA and (B) NHW.

Feature selection method	Features selected	Classification method	Classification accuracy (%)
(A)			
Boruta test—AA	2-Oxoisocaproic acid, arginine, a-tocopherol, citric acid, histidine, maltose, methionine, n-acetylglutamic acid, o-phosphoethanolamine, oxalic acid	Random forest	80.65
Boruta test—AA	2-Oxoisocaproic acid, arginine, a-tocopherol, citric acid, histidine, maltose, methionine, n-acetylglutamic acid, o-phosphoethanolamine, oxalic acid	Decision trees	79.03
Boruta test—AA	2-Oxoisocaproic acid, arginine, a-tocopherol, citric acid, histidine, maltose, methionine, n-acetylglutamic acid, o-phosphoethanolamine, oxalic acid	Logistic regression	77.42
Boruta test—AA	2-Oxoisocaproic acid, arginine, a-tocopherol, citric acid, histidine, maltose, methionine, n-acetylglutamic acid, o-phosphoethanolamine, oxalic acid	SVM	74.19
Recursive feature elimination—AA	Arginine, maltose, methionine, n-acetylglutamic acid, o-phosphoethanolamine	Random forest	79.03
Recursive feature elimination—AA	All features gave more accuracy than selecting some features	Decision trees	66.04
recursive feature elimination—AA	Arginine, maltose, methionine, n-acetylglutamic acid, o-Phosphoethanolamine	Logistic regression	74.19
Recursive feature elimination—AA	2-Oxoisocaproic acid, arginine, histidine, maltose, methionine, n-acetylglutamic acid, o-phosphoethanolamine, oxalic acid	SVM	75.81
(B)			
Boruta test—NHW	3-Hydroxybutanoic acid, 9,12-octadecadienoic acid, 9-hexadecenoic acid, a-ketoglutaric acid, b-alanine, cholesterol, citric acid, lactamide, oxalic acid, palmitic acid, p-cresol, pyruvic acid, tetradecanoic acid	Logistic regression	79.03
Boruta test—NHW	3-Hydroxybutanoic acid, 9-hexadecenoic acid, oxalic acid, palmitic acid, tetradecanoic acid	Random forest	75.61
Boruta test—NHW	3-Hydroxybutanoic acid, 9-hexadecenoic acid, cholesterol, oxalic acid, palmitic acid, tetradecanoic acid	Decision trees	60
Boruta test—NHW	3-Hydroxybutanoic acid, 9-hexadecenoic acid, cholesterol, oxalic acid, palmitic acid, tetradecanoic acid	SVM	61.73
Recursive feature elimination—NHW	3-Hydroxybutanoic acid, 9-hexadecenoic acid, oxalic acid, palmitic acid, tetradecanoic acid	Logistic regression	72.50
Recursive feature elimination—NHW	3-Hydroxybutanoic acid, 9-hexadecenoic acid, oxalic acid, palmitic acid, tetradecanoic acid	Random forest	75.61
Recursive feature elimination—NHW	3-Hydroxybutanoic acid, 9-hexadecenoic acid, oxalic acid, palmitic acid, tetradecanoic acid	Decision trees	60
Recursive feature elimination—NHW	3-Hydroxybutanoic acid, 9-hexadecenoic acid, oxalic acid, palmitic acid, tetradecanoic acid	SVM	62.86

The top features selected are shown.

### TCGA cohort mRNA and survival analyses

To gain molecular insights on the pathways altered in AA vs NHW individuals, the different abundances in AA vs NHW patients with breast cancer were mapped manually. Unpaired t-test was performed with Welch’s correction. To test the association of these metabolic genes with survival in AA breast cancer, we performed an analysis of the PanCancer atlas TCGA Cohort. The cohort was selected based on a cohort that contained information regarding self-reported race, and subtype. Cohort included AA cases (n = 82) and NHW cases (510) of ER+ breast cancer, that contained mRNA, protein expression and clinical outcomes.

### Ethical approval

UIC IRB Protocl #2017-1029.

## Results

### Race-specific plasma metabolic signatures

Study population included 102 patients with breast cancer (AA, n = 48; NHW, n = 54) and 148 healthy women as controls (AA, n = 100; NHW, n = 48). Demographic and clinical characteristics are shown in Table 1. Following data processing with minimum observed values for each metabolite, 83 metabolites were included in the analysis. For statistical purposes, we report statistical differences in p value < 0.05 obtained by t-tests. (Table 5).

**Table 5:** Significant features in AA and NHW according to statistical analysis: t-test.

	p value
Significant features in AA	
Arginine	0.0024
Methionine	0.005
Mimosine	0.012
2-Oxoisocaproic acid	0.0132
Citric acid	0.0307
Threonine	0.0378
Tyrosine	0.0389
Maltose	0.0393
Serine	0.0512
Proline	0.077
Tryptophan	0.078
Oxalic acid	0.0821
Significant features in NHW	
Palmitic acid	0.0001
9-Hexadecenoic acid	0.0001
3-Hydroxybutanoic acid	0.0003
Tetradecanoic acid	0.0005
9,12-Octadecadienoic acid	0.0012
P-Cresol	0.0013
Citric acid	0.0015
Pyruvic acid	0.003
Tryptophan	0.005
Oxalic acid	0.0068
Nicotinic acid	0.0177
Cysteine	0.0224
Lactic acid	0.0288
Tyrosine	0.0578
Glyceric acid	0.0799
Methionine	0.0874
Cholesterol	0.0962

### Aminoacids are good predictors of ER+ breast cancer in African American patients, while fatty acids are good predictors in Non-Hispanic White patients

**Figure 2:**
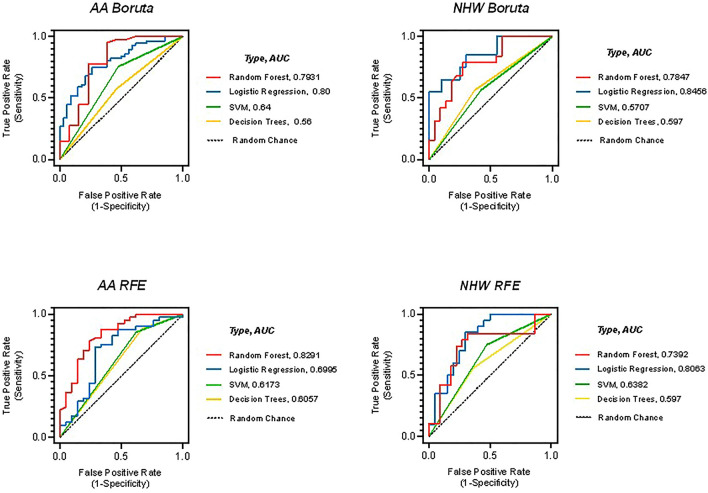
Feature selection and selection prediction method combinations identified top features that discriminate between cases and controls in AA and NHW.

We compared feature selection and prediction combinations instead of relying on only one machine learning method as previously described^39^. Boruta/Random Forest combination was the most accurate, or best combination of feature selection/prediction model for AA (AUC = 0.79, MCC = 0.56) and NHW (AUC = 0.78, MCC = 0.52), according to the Matthews correlation coefficient (MCC) (Fig. 2).

**Figure 3:**
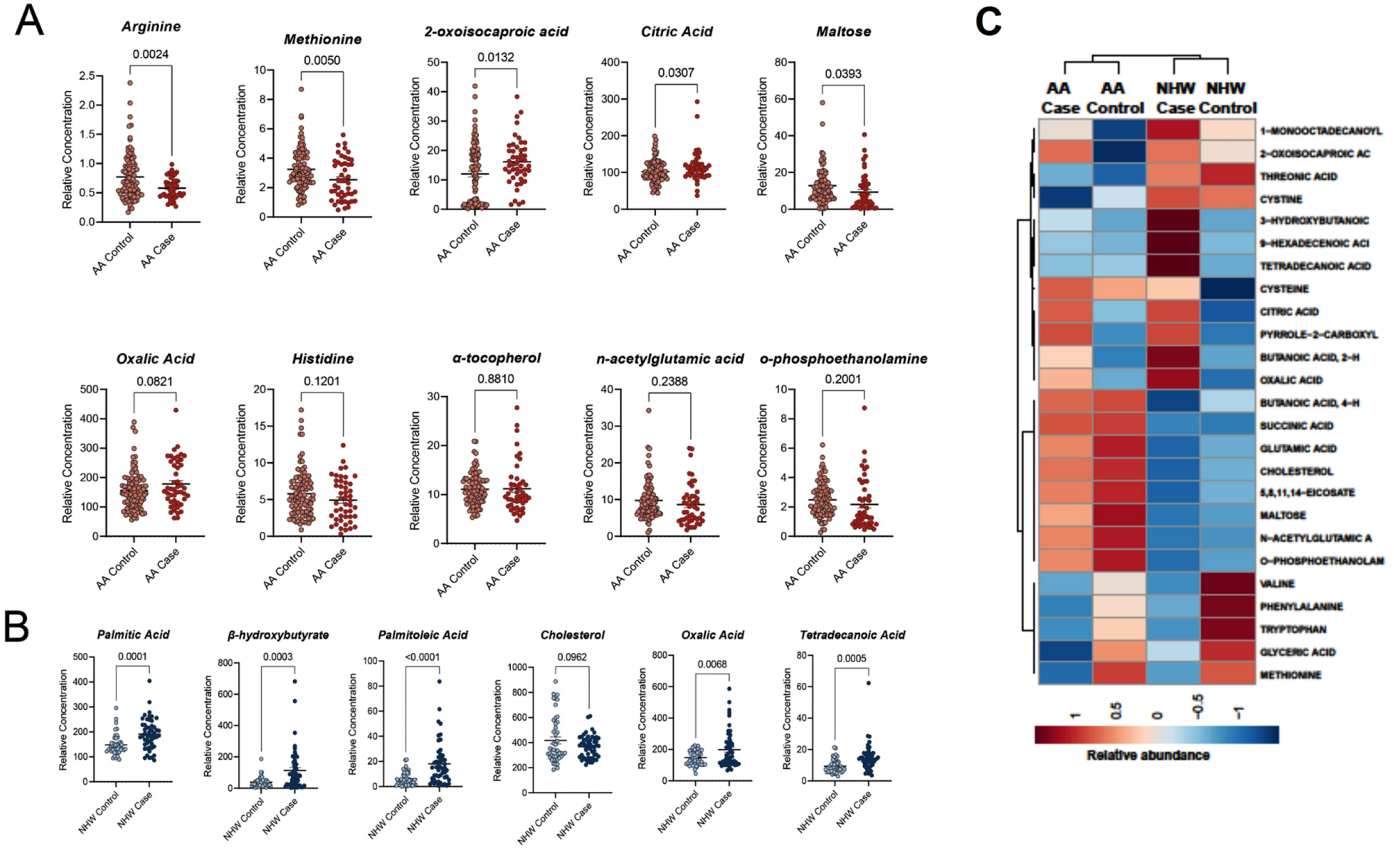
(**A**) Boxplot of the relative concentration of 10 differential metabolites in AA individuals divided by cancer cases and healthy controls. (**B**) Boxplot of the relative concentration of six differential metabolites in NHW individuals divided by cancer cases and healthy controls. The relative contribution of each feature towards the accuracy of predicting cancer cases is reported as mean Gini (**A**,**B**). The percentage of importance or relative influence that each feature contributes to the model are shown in the figure. (**C**) Heatmap showing metabolite levels in AA cases vs controls and NHW cases vs controls.

**Figure 4:**
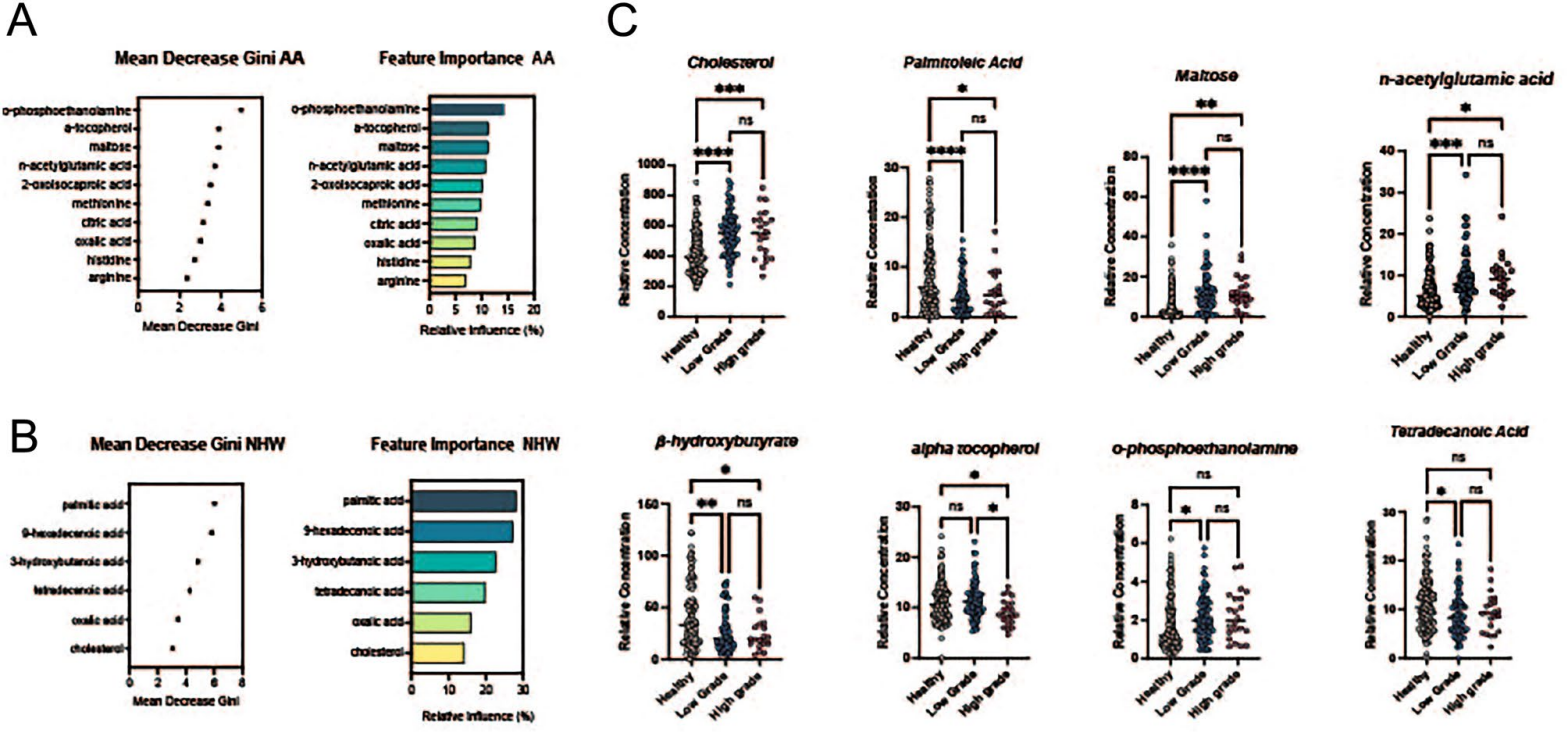
(**A**) Feature importance ranking with Mean Decrease Gini index calculated by the Random Forest (RF) algorithm for AA. The top features selected are shown. (**B**) Feature importance ranking with Mean Decrease Gini index calculated by the Random Forest (RF) algorithm for NHW The top features selected are shown. (**C**) Boxplot of relative concentration of 8 differential metabolites in cohort divided by healthy controls, low tumor grade and high tumor grade.

This optimal method identified 16 features that discriminate between cases and controls in both races. In AA individuals, a total of 10 features contributed to the signature to distinguish between case and controls and 9 of these features were exclusive for AA patients. These metabolites were: alpha ketoisocaproic acid, arginine, alpha tocopherol, citric acid, histidine, maltose, methionine, n-acetylglutamic acid, o-phosphoethanolamine, and oxalic acid. In AA individuals with breast cancer, we observed higher plasma levels of alpha ketoisocaproic acid a ketoacid, product of branched-chain aminoacid metabolism, alpha tocopherol oxalic acid, and citric acid, when compared to the healthy control group. In contrast, we observed lower arginine, histidine, maltose, methionine, n-acetyl glutamic acid, and o-phosphoethanolamine levels (Fig. 3A). In NHW individuals, a total of six features were identified as part of a signature that discriminates between case and controls: β-hydroxybutyrate, cholesterol, oxalic acid, palmitic acid, palmitoleic acid, and tetra decanoic acid. In NHW, we observed higher levels of all six metabolites in cases when compared with race-matched controls, and five of these features were exclusive for NHW patients (Fig. 3B,C). Overall, each of these differentially abundant features contributed to the metabolic signature significantly (Fig. 4A,B).

In order to evaluate the association of differential metabolite levels and tumor grade, a total of 148 breast cancer samples (with known tumor grade 1, 2, 3), were compared with 102 healthy controls. We grouped grades I and II breast cancer patients as “low grade” and grade III as “high grade” similar to previous studies^40–42^. We evaluated differential levels of all metabolites (n = 15) that were good predictors of breast cancer in Fig. 4A,B. Eight metabolites were found to be significantly changed among healthy controls and two grades of breast cancer (Fig. 4C).

### Key metabolic pathways activated in ER^+^ breast cancer patients differ by race

**Figure 5:**
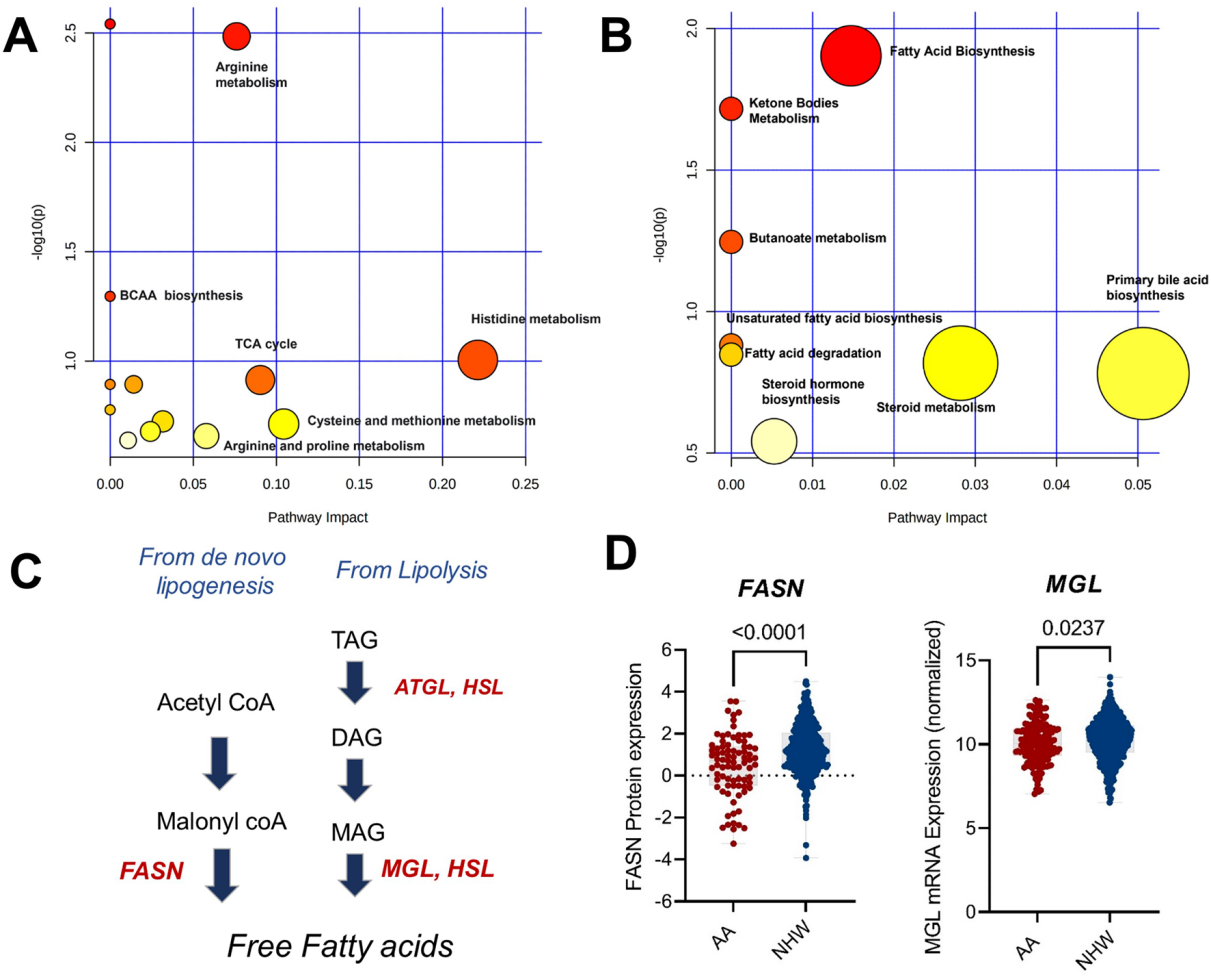
(**A**) Bubble plot showing metabolic pathways related to important features in African American individuals. The node color is based on its p value and the node radius is determined based on their pathway impact values. Pathway analysis performed with MetaboAnalyst 5.0. (**B**) Bubble plot showing Metabolic pathways related to important features in Non-Hispanic white individuals. The node color is based on its p value and the node radius is determined based on their pathway impact values. Pathway analysis performed with MetaboAnalyst 5.0. (**C**) Schematic of hypothesis in NHW patients, (**D**) protein levels of fatty acid synthase (FASN) and monoacylglycerol lipase (MGL) in NHW and AA patients with ER+ breast cancer.

Pathway-based analysis was performed to explore metabolic pathway differences according to race in breast cancer cases. For AA individuals, metabolites that are associated with aminoacyl-tRNA biosynthesis, arginine metabolism (p < 0.05), branched amino acid metabolism (p = 0.05), and histidine metabolism (p = 0.09) were differentially abundant in plasma from individuals with breast cancer (Fig. 5A). Other pathways including, TCA cycle, beta-alanine metabolism, sphingolipid metabolism, alanine, aspartate, and glutamate metabolism glyoxylate and dicarboxylate metabolism, cysteine and methionine metabolism, glycerophospholipid metabolism, arginine and proline metabolism, were not statistically significant, but were identified in the global pathway analysis.

In NHW individuals, three significant pathways were identified including fatty acid metabolism (p < 0.05), ketone body metabolism (p < 0.05), and butanoate metabolism (p = 0.05). Pathways like unsaturated fatty acid synthesis, fatty acid elongation and degradation, steroid metabolism, primary bile acid synthesis and steroid hormone synthesis were not statistically significant but were identified in the global pathway analysis (Fig. 5B). One potential explanation for this is that there is a higher production of free fatty acids coming from de novo lipogenesis or from triacylglycerol lipolysis. Therefore, we analyzed mRNA levels of key enzymes responsible for the generation of free fatty acids in tumors from AA vs NHW patients with ER+ breast cancer in TCGA. Expression of fatty acid synthase and Monoglycerol lipase was higher in tumors from NHW patients compared with AA patients (Fig. 5C,D).

### Metabolic pathway analysis suggests a role for epigenetic regulation differences that result in worse survival in African-American breast cancer patients

**Figure 6:**
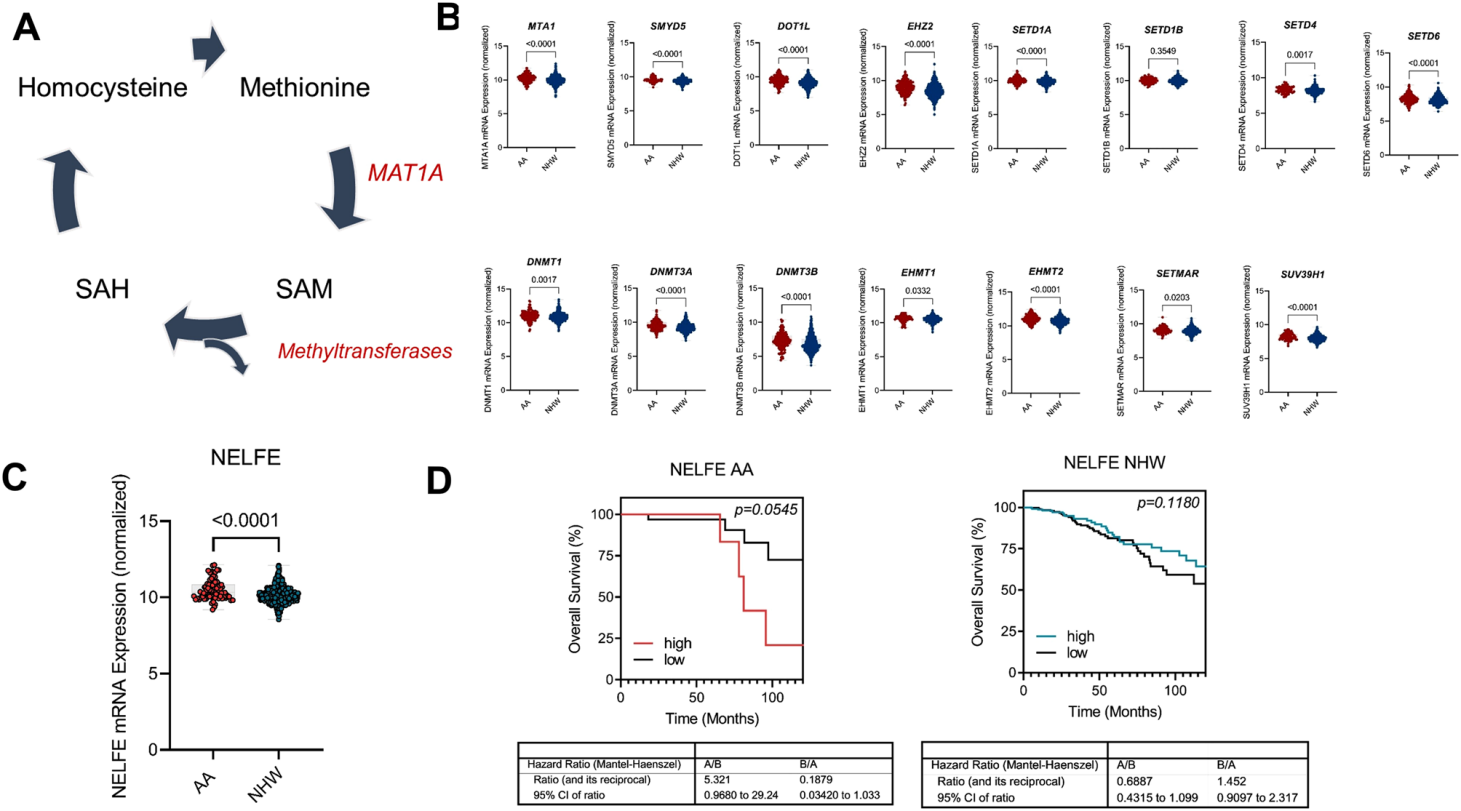
(**A**) Schematics of methionine metabolism to generate SAM. (**B**) Boxplot of mRNA levels of different DNA methyltransferases and histone methyl transferases. (**C**) Boxplots of mRNA levels of NELFE. (**D**) Kaplan Meier Curve sorted by low and high levels of NELFE in AA and NHW individuals.

In AA women with breast cancer, we observed lower methionine (Met) levels, which led us to hypothesize that there is a trend of higher demand for Met consumption and metabolism. One potential explanation for the utilization of Met is that it is being used as substrate for methylation processes. Therefore, we analyzed samples from Pan Cancer Atlas cohort (TCGA) for mRNA levels of MAT1A (methionine adenosyl transferase 1A) and methyltransferases (DNA and Histone) in AA (n = 183) compared with NHW individuals (n = 757). MAT1A is responsible for catalyzing the reaction from methionine to SAM. SAM then is used as a substrate for methyltransferases. We hypothesized and found that AA individuals have higher mRNA levels of methyl transferases and MAT1A. We identified 200 genes with histone methylation activity, and 71 genes with DNA methylation activity that were differentially expressed between AA and NHW individuals. Out of the 271 genes studied, 49 had increased expression in AA individuals, 15 of these were statistically significant (Fig. 6A). To obtain additional insights, we further investigated the associations of these genes with survival in AA and NHW breast cancer. A Kaplan Meier survival analysis of NELFE showed that high expression was associated with poor survival in AA women, but it was not associated with survival among NHW patients (Fig. 6B–D) Together these findings suggest that this gene may play role in the biology of breast cancer in AA individuals.

## Discussion

In this study, we established blood metabolite profiles of 102 patients with breast cancer and 148 healthy controls and we observed that metabolic profiles differed by race. We found that levels of amino acids were significantly lower in AA patients with breast cancer than healthy controls. One possible explanation might be the high demand for amino acids in breast tumor metabolism. Tumor cells require amino acids as alternative fuels, and as precursors of biosynthetic materials, including those for DNA synthesis, for building new blood vessels and supporting their rapid growth and proliferation. The alterations in these metabolic pathways cause differential activation of downstream signaling that further translate to altered oncogenic regulation. Breast cancer is associated with marked metabolic shifts. However, these shifts in metabolism within tumors can vary between stages, subtypes, and race^25,43,44^ Most differences involve variation in amino acid, fatty acids and TCA intermediates. Previous studies have shown that pathways associated with energy metabolism: glycolysis, amino acid metabolism and TCA cycle, are dominant in AA women with ER^+^ tumors, which potentially indicates the aggressiveness of their tumors^44,45^.

Additionally, changes in metabolite levels affect gene and protein expression by altering substrate availability of epigenetic modifiers that mark to the DNA, RNA, and histones, ultimately dictating cancer progression and outcome^46–48^. Previous studies^19–21, 49–51^ and studies from our lab^52–55^ showed how systemic or cellular metabolic changes impact epigenetic landscape, which is important for ERα activity and response to clinical drugs. Amino acids provide metabolic intermediates for epigenetic regulation. For example, carbon units from methionine cycle serve as a methyl donor for DNA and histone methyltransferases (DNMTs and HMTs). HMTs catalyze the transfer of methyl groups to lysine and arginine residues in histone proteins. Depending on the type of residue being modified, histone methylation regulates activation or repression of gene expression. In AA women, poverty levels correlate with hypermethylation of cancer-associated pathways including glucocorticoid receptor, p53, estrogen dependent breast cancer signaling, and cell proliferation^56^. Therefore, hypermethylation is a possible biological mechanism that can explain worse outcomes in AA women with breast cancer conferred by living in low socioeconomical status (SES) neighborhoods. We found that the methionine levels are lower in plasma samples from AA women with breast cancer. Methionine is a methyl group donor for methylation and represents the major contributor for epigenetic regulation. Both DNMTs and HMTs use S-adenosylmethionine (SAM) as a methyl donor, which is generated in the methionine cycle by the action of methionine adenolsyltransferase (MAT). Increased uptake of methionine leads to an excess of SAM, resulting in hypermethylation and aberrant histone methylation.

Aberrant hypermethylation in DNA can occur in promoter and enhancer regions of cancer related genes, such as tumor suppressor genes, resulting in loss of expression^57^. These abnormalities are involved in oncogenesis and tumor progression. Many cancer suppressor genes are silenced by DNA methylation in tissues^58^. A study in 2019 reported that residing in residential areas with high poverty may impact DNA methylation patterns. The authors reported that in AA women, poverty levels affected hypermethylation of important pathways including glucocorticoid receptor, p53, estrogen dependent breast cancer signaling, cell proliferation (BCL2, JUN, ESR1, ESR2, CYP19A1)^56^. Therefore, hypermethylation is a candidate biological mechanism that may contribute to worse outcomes in AA women with breast cancer because of a higher likelihood of residing in low SES neighborhoods.

Besides its function as methyl donor, SAM is involved in polyamine synthesis. Polyamines are essential for cell growth, and enzymes that catalyze this synthesis are often overexpressed in cancer. Studies have shown that polyamines can alter gene expression by modulating global chromatin structure and ultimately affecting cell proliferation^59,60^. Aminoacyl tRNAs (aa-tRNA) biosynthesis associated metabolites were enriched in AA individuals with breast cancer. The production of aminoacyl-tRNA is directly associated with the protein synthesis process. The main role of aa-tRNAs is to deliver the amino acid to the ribosome for incorporation into the polypeptide chain that is being produced during translation.

Moreover, we observed that the level of fatty acids in NHW patients with breast cancer was higher compared with healthy controls or AA individuals. These results might suggest higher rates of de novo lipogenesis or triacylglycerol lipolysis in the tumor. Highly proliferative cells use citrate produced in TCA cycle to generate fatty acids in the cytosol. The principal fatty acid synthesis enzyme, fatty acid synthase (FASN), is often upregulated in tumors^61^. Moreover, FASN has been detected in cell lysate and supernatant of breast cancer cells derived from NHW patients^62^. The regulation of de novo lipogenesis occurs mainly at the transcriptional level through the activation of sterol regulatory element binding proteins (SREBP). Its active N terminal fragment translocate to the nucleus and induces the transcription of gene that contain sterol regulatory elements (SRE) such as lipogenic enzymes (fatty acid synthase) FAS, ACLY (ATP citrate lyase) and ACC (acetyl coA carboxylase). In addition to its function in de novo lipogenesis, FASN is part of crosstalk between PI3K-AKT, MAPK-ERK2 pathways and their interaction with estradiol-ERα. The overexpression of lipogenic enzymes mentioned above, have been observed early in tumorigenesis. In ER+ cancer models, both genetic and pharmacological inhibition of FASN, hypersensitizes ERα to estrogen dependent transactivation, inducing estrogen receptor element (ERE) transcriptional activity and MAPK-ERK signaling^63^. Our results are consistent with previously published metabolomics studies that show fatty acids levels are higher in plasma from patients with breast cancer. In addition, our group previously reported that higher levels of free fatty acids in plasma were an indicator of high breast cancer risk, and result in mTOR and MAPK signaling and ERα recruitment to chromatin to increase transcriptional activity of factors that regulate cancer cell metabolism^53^. Consistently, other studies also reported a role for free fatty acids in other cancer types including lung^64^ childhood tumors^65^, and colon cancer^66^. Recent studies using biological deuterium fractionation and discrimination points out diet as the main source of increased fatty acid pool in plasma, which is delivered to cells via circulation. Thus, fatty acids act as the intermediate proton carrying carbon source for mitochondrial respiration. Further, generation of ketones using these deuterium-depleted fatty acids might explain benefit of ketogenic diets. Food insecurity and inequality in food quality and availability resulting in metabolic inefficiency might contribute to differential fatty acid profiles in AA vs. NHW women and breast cancer disparities.

One weakness of our study is the data imbalance. When data imbalance occurs, the number of samples in one class is larger than number of samples in the other class, and we can encounter an overoptimistic estimation of the classifier ability on the class with majority number of samples, such as our case (for AA individuals we have 100 healthy controls vs 48 cancer cases). To overcome this, we use the MCC score since produces a high score only if the prediction obtained good results in all the four confusion matrix categories (true positives, false negatives, true negatives, and false positives, Table 3). MCC score is a more reliable statistical rate and performance metric for binary classification when dealing with imbalanced data in binary data as it has been reported previously^67,68^.

In conclusion, our metabolic and bioinformatics analyses provided valuable information regarding metabolic alterations associated with cancer within each race, and provide insights on potential metabolic vulnerabilities that should be considered for depth studies of tumor biology that interrogate the role of candidate oncometabolites in breast cancer disparities, which may inform novel therapeutic development and biologically-informed epidemiologic studies to provide greater insight into biological mechanisms underlying racial disparities in breast cancer survival.

## Data Availability

The datasets generated and analyzed during the current study will be available from the corresponding author on reasonable request.

## Abbreviations

AA: African American
NHW: Non-Hispanic White
ER: Estrogen receptor
GC–MS: Gas chromatography–mass spectrometry
TCGA: The Cancer Genome Atlas
MAT1A: Methionine Adenosyl transferase
FASN: Fatty acid synthase
MGL: Monoacyl glycerol lypase
NELFE: Negative elongation factor
SES: Socioeconomical status
AJCC: American Joint Committee on Cancer
FDR: False discovery rate
AUC: Area under the curve
MCC: Matthews correlation coefficient
TCA: Tricarboxylic acid cycle
DNMTS: DNA methyl transferases
HMTS: Histone methyl transferases
SAM: S-adenosyl methionine
AATRNA: Aminoacyl-t-RNA
